# Residue Behaviors and Degradation Dynamics of Insecticides Commonly Applied to *Agrocybe aegerita* Mushrooms from Field to Product Processing and Corresponding Risk Assessments

**DOI:** 10.3390/foods13091310

**Published:** 2024-04-24

**Authors:** Qinghua Yao, Desen Su, Yunyun Zheng, Hui Xu, Minmin Huang, Meizhen Chen, Shaoxiao Zeng

**Affiliations:** 1 Institute of Quality Standards Testing Technology for Agro-Products, Fujian Key Laboratory of Agro-Products Quality and Safety, Fujian Academy of Agricultural Sciences, Fuzhou 350003, China; sudesen@faas.cn (D.S.); yyzheng@xmu.edu.cn (Y.Z.); hmmlwb@163.com (M.H.); mijing.meizhen@163.com (M.C.); 2 College of Food Science, Fujian Agriculture and Forestry University, Fuzhou 350002, China; xhuifst@163.com

**Keywords:** *Agrocybe aegerita* mushroom, pesticide residue, dissipation, processing, risk assessment

## Abstract

Residual pesticides in *Agrocybe aegerita* mushroom have emerged as a significant concern and bring much uncertainty due to processing procedures. In this study, a modified QuEChERS sample preparation procedure and UPLC-MS/MS were used to analyze the residual levels of four commonly used pesticides in *A. aegerita* from field to product processing. The field results showed that dissipation of these targeted chemicals was consistent with the first-order kinetics, and the half-life time ranged from 20.4 h to 47.6 h. The terminal residues of the four pesticides at harvest time ranged from 9.81 to 4412.56 μg/kg in raw mushroom. The processing factors (PFs) of clothianidin, diflubenzuron, chlorbenzuron, and pyridaben ranged from 0.119 to 0.808 for the drying process and from 0.191 to 1 for the washing process. By integrating the data from the field trials, the PFs, and the consumption survey, the chronic dietary risks of the target chemicals via *A. aegerita* intake ranged from 2.41 × 10^−5^ to 5.69 × 10^−2^ for children and from 6.34 × 10^−6^ to 1.88 × 10^−2^ for adults, which are considerably below the threshold of 1, indicating no unacceptable risk to consumers in the Fujian province of China. This research offers foundational data for appropriate use and the maximum residue limit (MRL) establishment for these four insecticides in *A. aegerita*.

## 1. Introduction

Owing to its extraordinary fragrance, affordable price, and high nutritional value, the black poplar mushroom (*A. aegerita*) has historically been one of the most consumed mushrooms in the United States and Asia [1,2], and it is commonly dry-processed before storage and consumption [3]. In the literature, a variety of biologically active ingredients, including agrocybenine with antifungal activity, lectins with antitumor activity, and indole derivatives with free radical scavenging activity, have been isolated from the fruiting body of *A. aegerita* [4,5,6]. Hence, *A. aegerita* could be regarded as a good source of bioactive peptides with numerous health benefits [2,7].

Unfortunately, *A. aegerita* yields are constantly affected by pathogens and insect-pests, such as *Mucor* spp., *Trichoderma* spp., *Aspergillus* spp., and *Mycetophila sciarid*, due to its long growth cycle and mild cultivation environment [8,9]. To combat these menaces, application of pesticides is not only common but essential during *A. aegerita* cultivation. On the basis of our previous field survey, there are approximately 50 pesticides belonging to several chemical families, such as organophosphates, organochlorine, pyrethrins, carbamates, benzoylureas, neonicotinoids, and dithiocarbamates, which may be practically used in the *A. aegerita* cultivation. However, as a minor mushroom variety, pesticide manufacturers have little interest in pesticide registration for *A. aegerita*. Up until now, no pesticides have been registered for *A. aegerita* cultivation in China. Consequently, many mushroom farmers resort to indiscriminate application of unregistered alternatives. Over the years, although surveillance schemes have been frequently conducted to monitor the pesticide residues on *A. aegerita*, the results only illustrate the detection frequencies and percentage of samples exceeding the maximum residue limits (MRLs), but they lack the information necessary for proper interpretation and objectification in terms of food safety [10]. Thus, more attention should be paid to the dietary exposure risks of these potential pesticide residues on *A. aegerita* products. Additionally, it is important to study the degradation dynamics of such pesticides, especially those with high efficiency and low toxicity, which are useful for future pesticide registration and the establishment of good agricultural practices (GAPs) related to *A. aegerita*.

Among the 50 pesticides practically used in *A. aegerita* cultivation, clothianidin, diflubenzuron, chlorbenzuron, and pyridaben show broad potential due to their outstanding properties, including a broad insecticidal spectrum, long-term control effect, and low toxicology [11,12,13,14]. It was reported that these four pesticides have been widely applied and rapidly grown worldwide [15,16,17,18]. However, comprehensive studies of the residues of clothianidin, diflubenzuron, chlorbenzuron, and pyridabenin on *A. aegerita* from field to product processing are scarce. In light of this, the major aims of this study were to (i) analyze the residues of these four pesticides in *A. aegerita* using a modified QuEChERS-UPLC-MS/MS method; (ii) compare the dissipation and terminal residue levels of the four pesticides in *A. aegerita* across field trials; (iii) determine the processing factors (PFs) associated with drying and washing processes; and (iv) assess the dietary risk posed by exposure to the corresponding pesticide residues for consumers based on the field trial data, PFs, and a previous mushroom consumption survey. The findings of this study hold immense practical and industrial significance.

## 2. Materials and Methods

### 2.1. Materials and Regents

The mycelia of *A. aegerita* were obtained from Lvhua Mushroom Co., Ltd. (Ningde, China). The standard solutions of clothianidin (CAS No. 210880-92-5, C_6_H_8_ClN_5_O_2_S, 99.8% purity), diflubenzuron (CAS No. 35367-38-5, C_14_H_9_ClF_2_N_2_O_2_, 99.0% purity), chlorbenzuron (CAS No. 57160-47-1, C_14_H_10_Cl_2_N_2_O_2_, 99.5% purity), and pyridaben (CAS No. 96489-71-3, C_19_H_25_ClN_2_OS, 98.5% purity) were obtained from Alta Scientific Co., Ltd. (Tianjin, China). HPLC-grade acetonitrile, ammonium formate, and formic acid were purchased from Merck (Darmstadt, Germany). Magnesium sulfate, sodium chloride, sodium citrate, and sodium citrate dibasic sesquihydrate were obtained from Shiyi Chemical Reagent Co., Ltd. (Shanghai, China). Ammonia solution was obtained from Aladdin Biochemical Technology Co., Ltd. (Shanghai, China). Primary secondary amine (PSA, 40–60 μm) was obtained from Sigma-Aldrich Corp. (St. Louis, MO, USA). Polytetrafluoroethylene (PTFE) filters (0.22 μm) were purchased from ANPEL Scientific Instrument Co., Ltd. (Shanghai, China). Ultrapure water (18.25 MΩ cm^−1^ resistivity) was prepared using a Direct-Q3 UV water purification system (Millipore Corporation, Burlington, MA, USA).

### 2.2. Field Trial Design and Sampling

The field trials were designed according to the Guidelines on Pesticide Residue Trials in China [19] with a minor modification and conducted between August and December of 2022 in an experimental house located at Gutian County (26°73′ N, 118°79′ E) in Fujian Province, China. During the field trials, the temperature and the relative humidity of the experimental house were maintained at 20–28 °C and 80–90%, respectively. In both the residue dynamic trials and the final residue trials, there were two treatment plots and one control plot for each targeted pesticide. Three replicates of a total area of 6 m^2^ were used in each plot, and a distance of 1 m was set between adjacent plots as a protection zone. The mycelia of *A. aegerita* were inoculated into a plastic bag pre-filled with sterilized substrates (seed hull 77%, wheat bran 20%, gesso powders 3%, and water content 52%). After 70 days of cultivation, the spraying treatment was designed, consisting of two dosage levels, 5 mL/m^2^ and 10 mL/m^2^. The application dosage and octanol–water partition coefficient (Logkow) of the pesticides are listed in Table 1. For the residue dynamic trials, each pesticide was sprayed once after diluting with 3.5 L of water when fruiting bodies of *A. aegerita* grew to an average height of 8 cm. A representative mushroom sample of 1 kg was randomly collected at 0 and 6 h and 1, 1.25, 2, 3, 4, and 5 d intervals after pesticide application. For the final residue trials, each pesticide was sprayed once on the substrate after diluting with 3.5 L of water, and fruiting began approximately 3 days thereafter. A representative sample was randomly collected at 3, 4, 5, and 6 d intervals. The collected samples were immediately ground with a grinder, stored in labeled polyethylene bottles, and kept in a freezer at −20 °C until analysis. 

### 2.3. Experiments with Processing Treatment

It is well known that processing treatment can affect the residual levels of pesticides. Typical processing operations employed for *A. aegerita* before consumption include drying and washing. Hence, another field trial of such pesticides should be performed for calculating the PFs. Based on the Guidelines from OECD [20], four targeted pesticides (30 mL of each) were mixed and diluted with 7 L of water and then sprayed on the fruiting bodies of an approximate height of 8 cm. The trial plot area was 12 m^2^. About 10 kg of mushroom sample was randomly collected at 8 h after pesticide application. Subsequently, samples were subjected to hot-air drying according to the actual production process [3]. At intervals of 0, 1, 3, 5, 7, 10, 13, 17, 20, and 24 h, *A. aegerita* was randomly collected, powdered with a grinder, and stored at −20 °C until analysis. 

For washing treatment, 20 g of dry mushroom sample was accurately weighed and rinsed with running tap water (1 L/min, 25 °C), immersed in 2 L of ultrapure water (25 °C) for 1 h, and finally ground and stored for further analysis. It is worth mentioning that the washing step used here corresponds closely to the actual conditions that are usually used in daily life. 

### 2.4. Extraction and Cleanup Procedure

The QuEChERS method is an efficient combination of solid- and liquid-phase extraction methods, which is a common sample preparation method for pesticide extraction [21]. Herein, a previous QuEChERS method [22] with minor modification was developed for extraction and cleanup of the targeted analytes in *A. aegerita*. First, 10 g of each fresh mushroom sample was weighed into a 50 mL PTFE centrifuge tube (for dry mushrooms, a 1.0× *g* sample was weighed into said centrifuge tube containing 9× mL of ultrapure water), and 10 mL of acetonitrile was added. The mixture was vortexed for 1 min; afterward, a total of 4.0 g of anhydrous MgSO_4_, 1 g of NaCl, 1 g of Na_3_C_6_H_5_O_7_, and 0.5 g of Na_2_HC_6_H_5_O_7_ was added and shaken for 5 min. The mixture was centrifuged for 5 min at 2400× *g* (Anke TDL-5-A, Shanghai, China). Following this, 6 mL of the upper layer was transferred into a centrifuge tube containing 900× mg of anhydrous MgSO_4_ and 150 mg of PSA. The resulting mixture was then vortexed for 1 min and centrifuged at 4400× *g* for 5 min. One milliliter of the supernatant was evaporated to dryness using a stream of nitrogen (40 °C). The residues were reconstituted in 1 mL of acetonitrile/water (5%, *v*/*v*) and then filtered with a 0.22 μm PTFE film for UPLC-MS/MS analysis.

### 2.5. UPLC-MS/MS Conditions

A Shimadzu Ultra-Fast Liquid Chromatography system comprising a solvent delivery system (Nexera X2 LC-30AD), an auto-sampler (Nexera X2 SIL-30AC), a degassing unit (DGU-20A_5R_), and a system controller (CBM-20A), equipped with a Shimadzu-8050 triple quadrupole mass spectrometer (Shimadzu, Kyoto, Japan), was used for instrumental analysis. The injection volume was 5 μL, and the chromatographic separation was achieved on an Acquity UPLCHSS T3 column with the following dimensions: 100 mm length, 2.1 mm ID, and 1.8 μm film thickness (Waters Corp., Milford, MA, USA). The mobile phase consisted of mobile phase A (Milli-Q water containing 5 mmol/L ammonium acetate and 0.1% formic acid) and mobile phase B (acetonitrile) at a flow rate of 0.3 mL min^−1^, with a gradient elution as follows: 0–1 min start with 10% B, 1–4 min from 10% to 50% B, 4–10 min from to 50% to 75% B, 10–12 min from 75% to 95% B, 12–17 min at 95% B, 17–17.1 min to 10% B, and 3.9 min equilibration time. With a 3.0 kV capillary voltage, the mass spectrometer operated in electron impact (EI) mode, and data were acquired using multiple reaction monitoring (MRM). The flow rate for drying the gas was set at 10 L/min, and the temperatures of the interface, desolvation, and heating block were set at 300 °C, 250 °C, and 400 °C, respectively.

### 2.6. Quality Control and Quality Assurance

In order to ensure the validation of the analytical methods, the parameters, including linearity, the matrix effect (ME), sensitivity, accuracy, and precision, were evaluated. Linearity was evaluated by analyzing the standard solution of concentration levels ranging from 0.2 μg/mL to 1000 μg/mL in triplicate. By comparing the slope of each matrix-matched calibration curve (S_m_) with the corresponding pure solvent calibration curve (S_s_) (ME = S_m_/S_s_) [23], the ME was assessed, and a value lower than 1 implies matrix effect suppression, while a value higher than 1 implies matrix effect enhancement. The limits of detection (LODs) and limits of quantification (LOQs) were defined as the analyte concentration based on a signal-to-noise ratio of 3 and the lowest spiked concentration with satisfactory recoveries of 70–120% with relative standard deviations less than 20%, respectively. Accuracy and precision were evaluated based on the recoveries and RSD of spiked experiments (three levels of standard solution were spiked into blank matrices with 5 replicates).

### 2.7. Statistical Analysis and Dietary Risk Assessment

#### 2.7.1. Dissipation Kinetics

The dissipation dynamics of pesticide residues were calculated using the first-order kinetics regression model, using Equation (1).

*C_t_* = *C*_0_ e^−*kt*^ (1)

where C_t_ is the residual level (mg/kg) at time t and C_0_ is the initial residual level (mg/kg). *k* is the dissipation coefficient (day^−1^), derived using the software sigma Plot 12.5 (Systat Software, San Jose, CA, USA). The half-lives (*t*_1/2_), the time required for the pesticide residual level to decrease to half of the initial residual level after application, were calculated using Equation (2).

*t*_1/2_ = ln (2)/*k* (2)

#### 2.7.2. Calculation of Processing Factors

The processing factor (PF) is defined as the ratio between the pesticide residual level (mg/kg) in the processed product to that (mg/kg) of the corresponding unprocessed (or raw) one [24]. It is calculated using the equation below:

*PF* = *R_after_*/*R_before_* (3)

A PF < 1 indicates a decrease in the residual level after processing, whereas a PF > 1 indicates an increase. When the residual level was below the LOD, it was considered not detected, and the value used to calculate was zero.

#### 2.7.3. Dietary Exposure Risk Assessment

Based on the data from the field trials, processing treatments, and previous mushroom consumption survey [3], the potential long-term dietary exposure risk via *A. aegerita* consumption was calculated according to Equations (4) and (5)

NEDI = F × STMR_i_ × PF_i_/bw (4)

cHQ = NEDI/ADI (5)

where NEDI (mg/kg bw per day) is the nationally estimated daily intake; STMR_i_ (mg/kg) is the median residual level in the field trial; F (kg/day) is the average edible mushroom intake per day; PF is the processing factor; bw (kg) is the average body weight; and ADI (mg/kg bw per day) is the acceptable daily intake. For clothianidin, diflubenzuron, chlorbenzuron, and pyridaben, it is 0.1, 0.02, 1.25, and 0.01 mg/kg bw per day, respectively [24]. cHQ is the chronic hazard quotient; a value of cHQ less than 1 indicates that the health risk posed by dietary exposure is acceptable. Otherwise, it is unacceptable.

## 3. Results and Discussion

### 3.1. Quality Control of Analytical Methods

**Figure 1:**
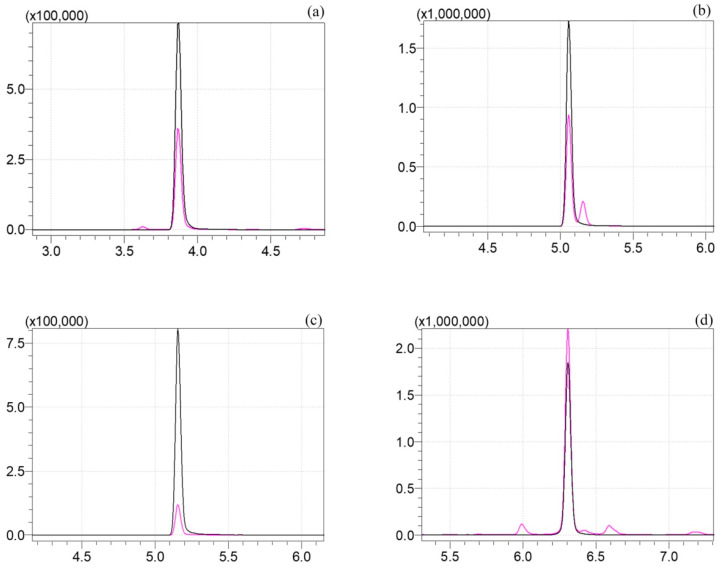
Chromatogram of clothianidin (**a**), diflubenzuron (**b**), chlorbenzuron (**c**), and pyridaben (**d**).

The analysis of pesticide residues is known to be difficult in such foods with complex components owing to matrix interference and complicated extraction procedures [25]. Due to high levels of polysaccharides, fiber, and other components in *A. aegerita*, the extraction, cleanup, and UPLC-MS/MS conditions for detection of clothianidin, diflubenzuron, chlorbenzuron, and pyridaben residues should be optimized. The cone voltages, collision energies, precursor, and product ions for targeted analytes are listed in Table 2. The mass spectrometer was operated in negative ionization mode for diflubenzuron and chlorbenzuron detection as well as in positive ionization mode for clothianidin and pyridaben. Figure 1 shows a representative chromatogram of clothianidin, diflubenzuron, chlorbenzuron, and pyridaben analyzed through UPLC-MS/MS with the optimized conditions. As it can be seen in Table 3, the ME of clothianidin was 0.61, which indicated ME suppression. On the contrary, ME enhancement was found in the case of diflubenzuron and chlorbenzuron. Additionally, there was no significant ME in pyridaben detection. Thus, the external matrix standards were applied for quantification of these pesticide residues to compensate for bias/interference. The assays of targeted analytes had good linearity with satisfying correlation coefficients (*R*^2^) higher than 0.999. The LODs of clothianidin, diflubenzuron, chlorbenzuron, and pyridaben were 0.15, 1.5, 0.15, and 0.03 μg/kg, respectively. The compound-specific LOQs were 0.50, 5, 0.50, and 0.10 μg/kg, respectively. Recoveries of these analytes spiked at three various concentrations ranging from 88.4% to 108.1% with RSDs from 3.4% to 4.8%. Therefore, the established method sufficiently met the requirements for the analysis of these four pesticide residues in real samples.

### 3.2. Dissipation and Terminal Residues of Four Pesticides in Fresh A. aegerita

**Figure 2:**
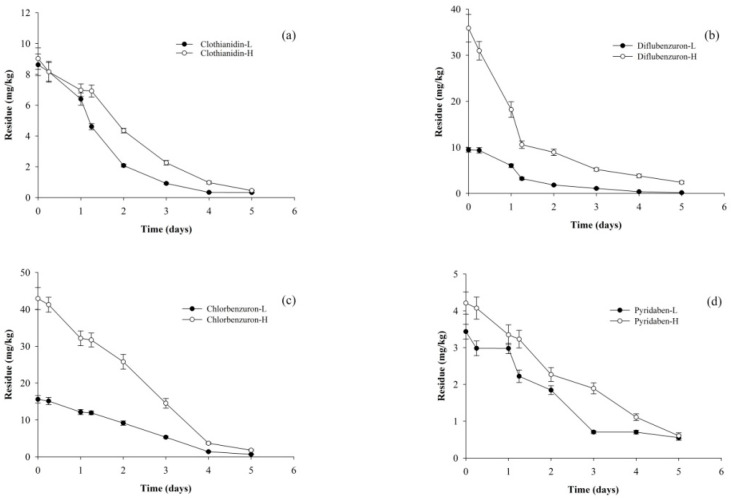
Dissipation trends of clothianidin (**a**), diflubenzuron (**b**), chlorbenzuron (**c**), and pyridaben (**d**) during field trials. (-L, low dosage; -H, high dosage).

The residue field trials involved spraying *A. aegerita* with 30% clothianidin Suspension Concentrate (SC), 20% diflubenzuron SC, 20% chlorbenzuron SC, and 15% pyridaben Emulsifiable Concentrate (EC), as described in Section 2.2. The initial deposited amount of clothianidin, diflubenzuron, chlorbenzuron, and pyridaben in *A. aegerita* at 2 h after spraying in the low-dosage group was 8.624 mg/kg, 9.449 mg/kg, 15.587 mg/kg, and 3.437 mg/kg, respectively, and 9.028 mg/kg, 35.884 mg/kg, 42.930 mg/kg, and 4.211 mg/kg, respectively, in the high-dosage group. The residual level gradually decreased over time, as depicted in Figure 2. The degradation trend of pesticides fit the first-order kinetics model well, as the regressive correlation coefficient (*R*) ranged from 0.9353 to 0.9878, and the corresponding half-lives of clothianidin, diflubenzuron, chlorbenzuron, and pyridaben were 22.9–31.9 h, 20.4–25.7 h, 31.1–31.3 h, and 40.3–47.6 h, respectively (Table 4). A plausible explanation is that their application concentrations, physicochemical properties, and commercial formulations were different. A similar phenomenon was found in the case of flonicamid and chlorfluazuron in tea gardens [26]. In addition, crop varieties, growth status, and climates can also cause inconsistencies in pesticide residues. Some published studies have reported that the half-life of chlorfluazuron was 6.03–8.10 d in cabbage, whereas it was 6.0 d in tea (Zhongcha 108) [26,27]. Additionally, the dissipation of bifenazate, etoxazole, fluazinam, lufenuron, and spirotetramat in raw citrus would be obviously much lower due to less rainfall [28]. To sum up, it is worth mentioning that compared to the dissipation of pesticides in other crops (i.e., tea, lettuce, or cauliflower) under open-field conditions, such pesticides in *A. aegerita* under culturing room conditions would inevitably degrade according to a special degradation trend.

For the final residue trials, the residues of four analytes in raw *A. aegerita* are shown in Table 5. The pesticide residues monitored in *A. aegerita* from the low-dosage group were 0.103–0.209 mg/kg for clothianidin, 0.058–0.205 mg/kg for diflubenzuron, 0.152 to 2.563 mg/kg for chlorbenzuron, and 0.010 to 0.287 mg/kg for pyridaben, respectively. The corresponding values in the high-dosage group for the four pesticides ranged from 0.104 to 0.145 mg/kg, 0.172 to 3.992 mg/kg, 0.442 to 4.412 mg/kg, and 0.058 to 1.171 mg/kg, respectively. The residual amounts decreased with increasing sampling intervals. Because the MRLs of clothianidin, chlorbenzuron, and pyridaben are not available for mushrooms under the national food safety standard of China [29], it could be only found that diflubenzuron residues in *A. aegerita* with enough sampling intervals were below the corresponding MRL (0.3 mg/kg). Thus, in order to guarantee the dietary safety and provide technical support for future GAP establishment, it is necessary to investigate whether the final residues pose an unacceptable health risk.

### 3.3. The Degradation Behavior of the Pesticides during Processing

**Figure 3:**
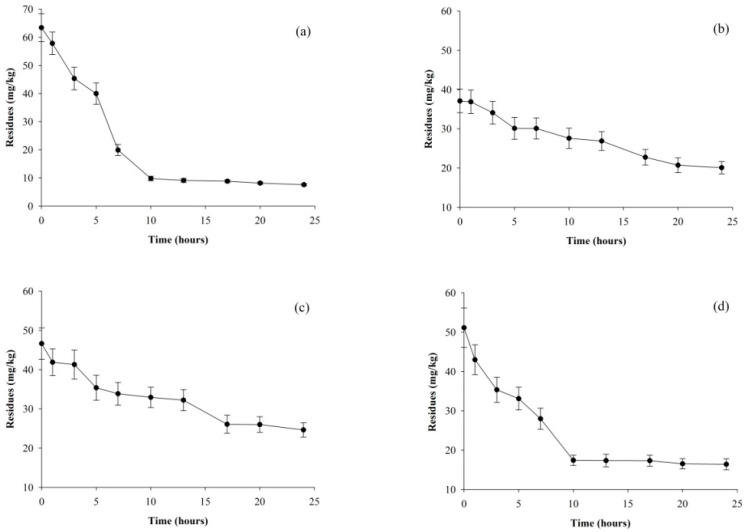
Dissipations trends of clothianidin (**a**), diflubenzuron (**b**), chlorbenzuron (**c**), and pyridaben (**d**) during the drying process.

As shown in Figure 3, it can be seen that the residues of four pesticides degraded according to different trends during the drying processes. In the initial 10 h, clothianidin and pyridaben exhibited a relatively higher dissipation rate, followed by a slight decrease. In contrast, diflubenzuron and chlorbenzuron dissipated gradually during the whole drying process. Actually, during the drying processes, pesticides may have dissipated not only due to loss through water evaporation from fresh mushrooms but also hydrolysis, oxidation, photolysis, co-distillation, and metabolism. The rate at which pesticides are moved and dissipated is closely related to the physico-chemical parameters of the pesticide themselves and the surrounding environmental conditions [30]. Hence, one possible reason could be that clothianidin and pyridaben are sensitive to high temperatures. As a thermal processing treatment, drying could be more effective for the breakdown of these two pesticides. For the washing process, it has comparatively no effect on reducing the residues of diflubenzuron and chlorbenzuron because of their extremely low solubility. Furthermore, diflubenzuron and chlorbenzuron exhibited similar change trends (Figure 3a,d).

**Figure 4:**
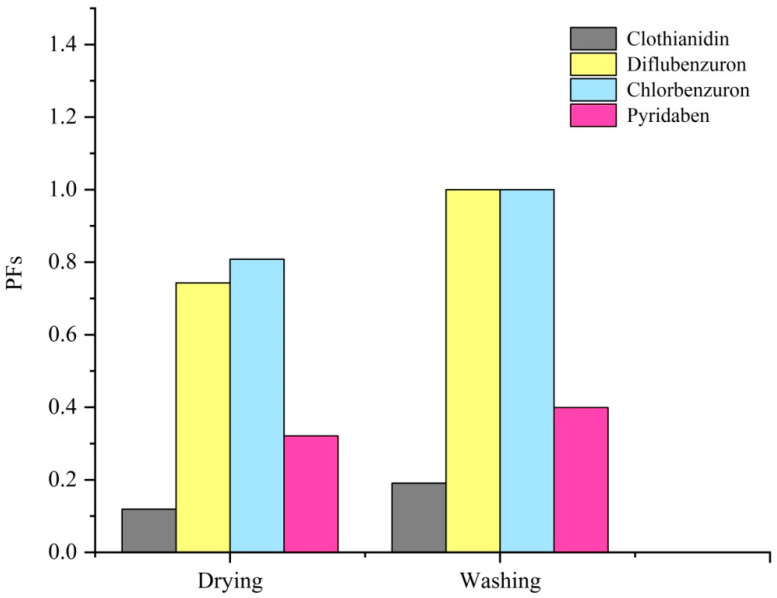
The processing factors of pesticides during drying and washing processes.

Routine combination and quantification of the processing factors into risk assessments would be helpful to provide a more realistic and clear picture of actual dietary exposure. In this study, PFs of four targeted pesticides are presented in Figure 4. For both drying and washing, it was indicated that the processing factors of diflubenzuron and chlorbenzuron were remarkably higher than those of clothianidin and pyridaben. To be precise, the PFs of the drying process for clothianidin, diflubenzuron, chlorbenzuron, and pyridaben are 0.119, 0.743, 0.808, and 0.321, respectively. For the washing process, the PFs are 0.191 for clothianidin, 1 for diflubenzuron, 1 for chlorbenzuron, and 0.399 for pyridaben.

### 3.4. Dietary Exposure Assessment

A clear dietary exposure assessment is important for informing risk management decisions and providing a realistic picture for final consumers [31]. Herein, the chronic health risks of long-term dietary exposure to pesticide residues in *A. aegerita* for adults and children are illustrated in Table 5. On the whole, the chronic hazard quotient (cHQ) values for all targeted pesticides were far below 1. This indicated that the exposure risk is was acceptable upon *A. aegerita* consumption in the Fujian province of China. Based on consideration of PFs, the cHQ of fresh mushroom consumption was much higher than that of dry mushrooms, especially for clothianidin and pyridaben. This also meant that household or industrial processing was effective in reducing the pesticide exposure risk levels via food intake. Additionally, for different consumer subgroups, children usually have higher dietary exposure risk than adults due to their relatively higher mushroom intake per kg of body weight. Such a phenomenon has been found among many food items [32,33,34]. Our findings align with these previous studies. Indeed, it is necessary to emphasize that children are more sensitive to undesirable chemicals due to their immature defense systems. Therefore, a national surveillance program of foods is of tremendous significance even if GAPs are established.

Finally, some uncertainties involved in the risk assessment of this study should be acknowledged. Firstly, except for drying and washing, the PFs of other household processes (i.e., boiling and frying) were assigned as 1 to this study, which may lead to over-estimated risks. Huan et al. [35] reported that frying and stir-frying were effective at reducing residues of pesticides with high *K_ow_* in cowpea. Oliva et al. [36] found that pyriproxyfen and deltamethrin in zucchini significantly decreased after washing, blanching, and freezing. Secondly, although various measures (i.e., using mushroom images to assist consumers in estimating food intake or increasing the number of surveyed participants) were taken to reduce uncertainties in our mushroom consumption survey, there still remains some level of uncertainty due to self-reported parameters, such as body weight. This is often handled by including more participants and more recording days, but this is an expensive approach. Again, no method can perfectly reflect true food intake without uncertainty [37]. Moreover, because we conducted the risk assessment only based on a deterministic approach in this study, uncertainties may arise from insufficient knowledge about exposure scenarios and models. However, considering that the RQ values calculated using the deterministic approach are extremely low, it is not necessary to conduct a further risk assessment via the probabilistic approach [38]. Another concern that should be stated is that we only present the pesticide exposure risk from *A. aegerita*; as such, a more complete assessment of all dietary mushrooms should be performed in the future when more data are available.

## 4. Conclusions

Our study constitutes the first to comprehensively describe the dissipation trends of four pesticides (clothianidin, diflubenzuron, chlorbenzuron, and pyridaben), which are commonly applied in *A. aegerita* cultivation, under field trial and processing conditions and then assess the chronic dietary exposure risks. With the establishment of a reliable and robust method for pesticide determination, the half-lives of pesticides in *A. aegerita* under field trials were calculated as 22.9–31.9 h for clothianidin, 20.4–25.7 h for diflubenzuron, 31.1–31.3 h for chlorbenzuron, and 40.3–47.6 for pyridaben. Clothianidin and pyridaben exhibited significant reductions of 88.1% and 67.9% in the drying step, as well as 80.9% and 60.1% in the washing step, owing to their sensitivity to high temperatures and high solubility. However, chlorfluazuron and diflubenzuron residues largely persisted in *A. aegerita*. Based on a deterministic risk assessment approach, the RQ values of these four pesticide residues were far less than 1, indicating that the potential risks posed by targeted undesirable chemicals via *A. aegerita* consumption were acceptable. Nevertheless, caution should be taken for children, as their RQ values were significantly higher than those of adults due to their relatively higher daily mushroom intake per kilogram of body weight. In summary, despite these pesticides not currently being registered for *A. aegerita* cultivation, these results may offer useful and valuable insights when establishing future GAPs and when setting corresponding MRLs to protect consumer health.

## Figures and Tables

**Table 1:** Application dosage (g a.i./m^2^) and Logkow of the pesticides in the field trials for *A. aegerita.*

Pesticides	Logkow	Low Dosage	High Dosage	Control
Diflubenzuron	3.89	1	2	0
Clothianidin	0.7	0.75	1.5	0
Chlorbenzuron	/	1	2	0
Pyridaben	6.37	0.75	1.5	0

**Table 2:** The MS/MS parameters for the detection of clothianidin, diflubenzuron, chlorbenzuron, and pyridaben.

Compound	Retention Time (min)	Ion Pairs (*m/z*)	Cone Voltage/V	Collision Energy (eV)
Clothianidin	4.247	250.00 > 169.00 ^a^	−11.0	−10.0
		250.00 > 131.85	−11.0	−14.0
Diflubenzuron	7.812	309.10 > 289.05 ^a^	21.0	8.0
		309.10 > 156.15	21.0	10.0
Chlorbenzuron	8.174	307.00 > 154.15 ^a^	21.0	11.0
		307.00 > 126.15	21.0	22.0
Pyridaben	12.606	365.10 > 309.05 ^a^	−18.0	−12.0
		365.10 > 147.10	−18.0	−25.0

^a^, quantitative ion pairs.

**Table 3:** The linear regression equation, MEs, LODs, LOQs, recovery rate, and RSD for the targeted analytes obtained through UPLC-MS/MS determination.

Compound	Linear Range (μg/mL)	Equation	*R* ^2^	MEs	LOD (μg/kg)	LOQ (μg/kg)	Recovery (%)	RSD (%)
Clothianidin	1–100	y = 43,910x − 16,764	0.9995	0.61	0.15	0.50	97.2	4.6
Diflubenzuron	10–1000	y = 4289x − 16,209	0.9996	1.83	1.5	5	88.4	4.8
Chlorbenzuron	1–100	y = 20,681x − 6505	0.9997	1.48	0.15	0.50	100.4	3.4
Pyridaben	0.2–20	y = 263,273x + 129,006	0.9994	1.00	0.03	0.10	108.1	4.1

*R*^2^, correlation coefficient. MEs, matrix effects. LOD, limit of detection. LOQ, limit of quantification. RSD, relative standard deviation.

**Table 4:** Kinetic models and half-lives of four targeted analytes.

Group	Compound	Regression Equation	*R*	Half-Life (h)
Low dosage	Clothianidin	Ct = 9.7163 × 10^−0.7273x^	0.9815	22.9
	Diflubenzuron	Ct = 10.3833 × 10^−0.8166x^	0.9871	20.4
	Chlorbenzuron	Ct = 18.2120 × 10^−0.5319x^	0.9622	31.3
	Pyridaben	Ct = 3.5790 × 10^−0.4129x^	0.9353	40.3
High dosage	Clothianidin	Ct = 10.1223 × 10^−0.5214x^	0.9766	31.9
	Diflubenzuron	Ct = 33.6787 × 10^−0.6483x^	0.9748	25.7
	Chlorbenzuron	Ct = 49.6793 × 10^−0.5350x^	0.9622	31.1
	Pyridaben	Ct = 4.5355 × 10^−0.3494x^	0.9878	47.6

**Table 5:** Risk assessment of exposure to four insecticides due to *A. aegerita* consumption.

Group	Compound	Residues (μg/kg)				cHQ of Fresh Samples		cHQ of Dry Samples	
		Min	HR-P	STMR	Average	Adults	Children	Adults	Children
Low	Clothianidin	103.04	209.11	131.74	151.93	7.30 × 10^−4^	2.20 × 10^−3^	6.34 × 10^−6^	2.41 × 10^−5^
	Diflubenzuron	58.29	2047.50	321.36	568.54	8.90 × 10^−3^	2.69 × 10^−2^	2.53 × 10^−3^	9.62 × 10^−3^
	Chlorbenzuron	151.64	2562.94	284.95	817.86	1.26 × 10^−4^	3.81 × 10^−4^	3.90 × 10^−5^	1.48 × 10^−4^
	Pyridaben	9.81	287.17	68.78	103.48	3.81 × 10^−3^	1.15 × 10^−2^	1.86 × 10^−4^	7.10 × 10^−4^
High	Clothianidin	104.42	1447.04	214.22	529.72	1.19 × 10^−3^	3.58 × 10^−3^	1.03 × 10^−5^	3.92 × 10^−5^
	Diflubenzuron	171.99	3991.58	680.12	1337.81	1.88 × 10^−2^	5.69 × 10^−2^	1.36 × 10^−5^	5.20 × 10^−5^
	Chlorbenzuron	441.71	4412.56	719.33	1468.91	3.19 × 10^−4^	9.63 × 10^−4^	9.84 × 10^−5^	3.74 × 10^−4^
	Pyridaben	58.32	1171.42	200.31	431.14	1.11 × 10^−2^	3.35 × 10^−2^	5.43 × 10^−4^	2.07 × 10^−3^

cHQ, the chronic hazard quotient. HR-P, the highest residual levels. STMR, the median residual levels.

## Data Availability

The original contributions presented in this study are included in the article; further inquiries can be directed to the corresponding authors.

## References

[1] Lin S., Ching L., Lam K., Cheug P.C.K. (2017). Anti-angiogenic effect of water extract from the fruiting body of *Agrocybe aegerita*. LWT Food Sci. Technol..

[2] Song R., Liang T., Shen Q., Liu J., Lu Y., Tang C., Chen X., Hou T., Chen Y. (2020). The optimization of production and characterization of antioxidant peptides from protein hydrolysates of *Agrocybe aegerita*. LWT-Food Sci. Technol..

[3] Yao Q., Su D., Huang M., Zheng Y., Chen M., Lin Q., Xu H., Zeng S. (2024). Residue degradation and metabolism of dinotefuran and cyromazine in *Agrocybe aegerita*: A risk assessment from cultivation to dietary exposure. J. Food Compos. Anal..

[4] Landi N., Pacifico S., Ragucci S., Di Giuseppe A., Iannuzzi F., Zarrelli A., Piccolella S., Di Maro A. (2017). Pioppino mushroom in southern Italy: An undervalued source of nutrient and bioactive compounds. J. Sci. Food Agric..

[5] Ragucci S., Landi N., Russo R., Valletta M., Citores L., Iglesias R., Pedone P., Pizzo E., Di Maro A. (2020). Effect of an additional N-terminal methionyl residue on enzymatic and antifungal activities of Ageritin purified from *Agrocybe aegerita* fruiting bodies. Int. J. Biol. Macromol..

[6] Li G., Liu X., Cong S., Deng Y., Zheng X. (2021). A novel serine protease with anticoagulant and fibrinolytic activities from the fruiting bodies of mushroom *Agrocybe aegerita*. Int. J. Biol. Macromol..

[7] Diyabalanage D., Mulabagal V., Mills G., DeWitt D.L., Nair M.G. (2008). Health-beneficial qualities of the edible mushroom, *Agrocybe aegerita*. Food Chem..

[8] Choi I.Y., Choi J.N., Sharma P.K., Lee W.H. (2010). Isolation and identification of mushroom pathogens from *Agrocybe aegerita*. Mycobiology.

[9] Jiao J., Shi Y.C., Wu D. (2019). Residual dynamic of imidacloprid in *Agrocybe aegerita* and its substrates. Agrochemistry.

[10] Łozowicka B., Kaczyński P., Jankowska M., Rutkowska E., Hrynko I. (2012). Pesticide residues in raspberries (*Rubus idaeus* L.) and dietary risk assessment. Food Addit. Contam. Part B.

[11] Sales-Alba A., Cruz-Alcalde A., López-Vinent N., Cruz L., Sans C. (2023). Removal of neonicotinoid insecticide clothianidin from water by ozone-based oxidation: Kinetics and transformation products. Sep. Purif. Technol..

[12] Dai P., Jack C.J., Mortensen A.N., Bloomquist J.R., Ellis J.D. (2018). The impacts of chlorothalonil and diflubenzuron on *Apis mellilera* L. larvae reared in vitro. Ecotoxicol. Environ. Saf..

[13] Zhao Y., Zou C., Zhang L., Li C., Li X., Song L. (2023). Chlorbenzuron caused growth arrest through interference of glycolysis and energy metabolism in *Hyphantria cunea* (Lepidoptera: Erebidae) larvae. Pestic. Biochem. Phys..

[14] Han Y., Dong F., Xu J., Liu X., Li Y., Kong Z., Li Y., Kong Z., Liang X., Liu N. (2014). Residue change of pyridaben in apple samples during apple cider processing. Food Control.

[15] You T., Ding Y., Chen H., Song G., Huang L., Wang M., Hua X. (2022). Development of competitive and noncompetitive immunoassays for clothianidin with high sensitivity and specificity using phage-displayed peptides. J. Hazard. Mater..

[16] Aguilera A., Valverde A., Camacho F., Boulaid M., García-Fuentes L. (2014). Household processing factors of acrinathrin, fipronil, kresoxim-methyl and pyridaben residues in green beans. Food Control.

[17] Chang J., Wang H., Xu P., Guo B., Li J., Wang Y., Li W. (2018). Oral and dermal disturbance in the Mongolian racerunner (*Eremias argus*). Environ. Pollut..

[18] Yang X., Luo J., Li S., Liu C. (2016). Evaluation of nine pesticide residues in three minor tropical fruits from southern China. Food Control..

[19] (2018). Agricultural Industry Standard of China—Guideline on Pesticide Residue Trial.

[20] OECD—Organization for Economic Co-operation and Development (2007). OECD Guideline for Testing of Chemicals, No. 508.

[21] Arani H.M., Kermani M., Kalantary R.R., Jaafarzadeh N., Arani S.B. (2023). Pesticides residues determination and probabilistic health risk assessment in the soil and cantaloupe by Monte Carlo simulation: A case study in Kashan and Aran-Bidol, Iran. Ecotoxicol. Environ. Saf..

[22] (2021). National Food Safety Standard—Determination of 331 Pesticides and Metabolites Residues in Foods of Plant Origin-Liquid Chromatography-Tandem Mass Spectrometry Method.

[23] Li Y., Xu J., Zhao X., He H., Zhang C., Zhang Z. (2021). The dissipation behavior, house hold processing factor and risk assessment for cyenopyafen residues in strawberry and mandarin fruits. Food Chem..

[24] Mekonen S., Ambelu A., Spanoghe P. (2015). Effect of household coffee processing on pesticide residues as a means of ensuring consumers’ safety. J. Agric. Food Chem..

[25] Kanrar B., Mandal S., Bhattacharyya A. (2010). Validation and uncertainty analysis of a multiresidue method for 42 pesticides in made tea, tea infusion and spent leaves using ethyl acetate extraction and liquid chromatography-tandem mass spectrometry. J. Chromatogr. A.

[26] Li H., Zhong Q., Wang X., Luo F., Zhou L., Sun H., Yang M., Luo Z., Chen Z., Zhang X. (2021). The degradation and metabolism of chlorfluazuron and flonicamid in tea: A risk assessment from tea garden to cup. Sci. Total Environ..

[27] Ganguly P., Barik S.R., Patra S., Roy S., Bhattacharyya A. (2017). Persistence of chlorfluazuron in cabbage under different agro-climatic conditions of India and its risk assessment. Environ. Toxicol. Chem..

[28] Tang H., Sun Q., Huang J., Wen G., Han L., Wang L., Zhang Y., Dong M., Wang W. (2023). Residue behaviors, degradation, processing factors, and risk assessment of pesticides in citrus from field to product processing. Sci. Total Environ..

[29] (2021). National Food Safety Standard—Maximum Residue Limits for Pesticides in Food.

[30] Bajwa U., Sandhu S.K. (2011). Effect of handling and processing on pesticide residues in food—A review. J. Food Sci. Technol..

[31] Kettler S., Kennedy M., McNamara C., Oberdörfer R., O’Mahony C., Schnabel J., Smith B., Sprong C., Faludi R., Tennant D. (2015). Assessing and reporting uncertainties in dietary exposure analysis mapping of uncertainties in a tiered approach. Food Chem. Toxicol..

[32] Yao Q., Yan S., Chen H., Li J., Lin Q. (2020). Dietary risk assessment of pesticide residues on *Tremella fuciformis* Berk (snow fungus) from Fujian Province. Food Addit. Contam. Part A.

[33] Cui K., Wu X., Zhang Y., Cao J., Wei D., Xu J., Dong F., Liu X., Zheng Y. (2021). Cumulative risk assessment of dietary exposure to triazole fungicides from 13 daily-consumed foods in China. Environ. Pollut..

[34] Cui K., Guan S., Liang J., Fang L., Ding R., Wang J., Li T., Dong Z., Wu X., Zheng Y. (2023). Dissipation, metabolism, accumulation, processing and risk assessment of fluxapyroxad in cucumber and cowpea vegetables from field to table. Food Chem..

[35] Huan Z., Xu Z., Jiang W., Chen Z., Luo J. (2015). Effect of Chinese traditional cooking on eight pestcides residue during cowpea processing. Food Chem..

[36] Oliva J., Cermeño S., Cámara M.A., Martínez G., Barba A. (2017). Disappearance of six pesticides in fresh and processed zucchini, bioavailability and health risk assessment. Food Chem..

[37] EFSA (2006). Guidance of the Scientific Committee on a request from EFSA related to Uncertainties in Dietary Exposure Assessment. EFSA J..

[38] Claeys W.L., Schmit J., Bragard C., Maghuin-Rogister G., Pussemier L., Schiffers B. (2011). Exposure of several Belgian consumer groups to pesticide residues through fresh fruit and vegetable consumption. Food Control.

